# Pharmaceutical care for patients with breast and ovarian cancer

**DOI:** 10.1007/s00520-012-1385-z

**Published:** 2012-02-02

**Authors:** Andrea Liekweg, Martina Westfeld, Michael Braun, Oliver Zivanovic, Tania Schink, Walther Kuhn, Ulrich Jaehde

**Affiliations:** 1 Institute of Pharmacy, Department of Clinical Pharmacy, University of Bonn, An der Immenburg 4, 53121 Bonn, Germany; 2 Center for Integrated Oncology (CIO), Department of Obstetrics and Gynaecology, University of Bonn, Sigmund-Freud-Str. 25, 53127 Bonn, Germany; 3 Department of Clinical Epidemiology, Bremen Institute for Prevention Research and Social Medicine, Achterstr. 30, 28359 Bremen, Germany

**Keywords:** Breast cancer, Patient-reported outcomes, Pharmaceutical care, Quality of life, Toxicity, Supportive care

## Abstract

**Purpose:**

Individualized patient care may help reduce the incidence of adverse drug events in systemic cancer therapy. This study was conducted to explore the feasibility and potential of additional pharmaceutical care for breast and ovarian cancer patients.

**Methods:**

The study was designed as a prospective, multicentered cohort study with a control group. Ninety-eight breast or ovarian cancer patients were recruited from outpatient oncology clinics and primary care oncologists: initially into the control group receiving standard care and after implementation of pharmaceutical care into the intervention group consisting of additional patient counseling on the management of treatment-associated adverse events and optimization of supportive medication. Primary outcome was the complete response to the antiemetic prophylaxis. Secondary endpoints were the severity of nausea, frequency of emesis, health-related quality of life, and patient satisfaction with information on cancer treatment education.

**Results:**

Forty-eight patients were included in the control group and 50 in the intervention group. Of the patients, 35.4% in the control group and 76.0% in the intervention group (*p* < 0.001) had a complete response to the antiemetic prophylaxis. The severity of acute and delayed nausea was not reduced. The global health scale and two symptom scales (nausea and vomiting, appetite loss) of the EORTC QLQ-C30 questionnaire were positively affected by pharmaceutical care. Patient satisfaction with information was significantly higher in the intervention group.

**Conclusions:**

Patients with breast and ovarian cancer seem to benefit from pharmaceutical care, as suggested by improved patient-reported outcomes such as emetic episodes, quality of life, and patient satisfaction after implementation.

**Electronic supplementary material:**

The online version of this article (doi:10.1007/s00520-012-1385-z) contains supplementary material, which is available to authorized users.

## Introduction

Therapeutic strategies for cancer patients are highly individualized and include a variety of drugs with different pharmacological mechanisms and targets. As anticancer therapy is often associated with severe adverse drug events, there is an increasing demand for effective supportive care strategies preventing or ameliorating drug-induced toxicity. In the last decade, several evidence-based clinical practice guidelines have been developed for supportive care. Still, adverse drug events range high among the most feared consequences associated with antineoplastic therapy [1]. Chemotherapy-induced nausea and vomiting is considered to be substantially distressing for the patients and should be addressed by oncology care services [2]. However, Mertens et al. showed that physicians do not always adhere adequately to antiemetic prescribing guidelines, indicating that the sole distribution of guidelines does not lead to a better control of nausea and vomiting. The situation improved after nurse practitioner antiemetic prescribing was introduced [3].

It is widely accepted that multidisciplinary care models have a high potential to enhance patient safety [4]. Considering the fact that patients with solid tumors are mainly treated in outpatient settings, structured patient counseling on their individual chemotherapy including medication reconciliation may be of particular benefit to patients experiencing transitions in care [5]. Pharmaceutical care is a concept, which may contribute to this approach. It is defined as the responsible provision of drug therapy for the purpose of achieving definite outcomes that improve a patient’s quality of life [6]. The addition of a pharmacist to the health care team may ensure appropriate medication use and maximize adherence, as recently stated in a NCCN Task Force Report [7]. As antineoplastic drug therapy follows established protocols, pharmaceutical care models in oncology aim at minimizing treatment-related toxicity and, therefore, focus on optimizing supportive care strategies [8]. Moreover, they include individualized patient information on adverse events as this is of great importance for many patients [9]. Different coping strategies are associated with different needs regarding amount and depth of information [10]. Therefore, patient satisfaction and the capability to initiate self-care behavior depend on the quality of individually tailored information.

New treatment strategies and care models are increasingly evaluated by means of patient-reported outcomes (PROs) [11]. In oncology, self-reporting of toxicity symptoms has successfully been established, which may provide some advantages over reporting by clinicians [12]. The aim of this study was to explore the feasibility and potential of pharmaceutical care for breast and ovarian cancer patients treated in outpatient settings in Germany by measuring PROs. The pharmaceutical care model included the use of supportive medication according to evidence-based treatment guidelines and patient counseling on the management of treatment-associated adverse events.

## Methods

### Patients and setting

Six academic- and community-based outpatient clinics as well as two primary care oncologists in West and North Germany participated in the study. Patients with a diagnosis of breast or ovarian cancer receiving their first chemotherapy were consecutively recruited in each center. All standard adjuvant and neoadjuvant chemotherapy regimens for the treatment of breast and ovarian cancer were allowed. Patients had to be at least 18 years of age, give their written informed consent, and be able to speak, read, and write German. Patients were excluded from the study if they had diseases or mental states which impeded that the patient completely understood the provided information on the study or if the patient had an impaired capability of reading and completing questionnaires self-administered. Written informed consent was obtained from all participating patients prior to any study-related procedures.

### Study design

The study was conducted as a prospective, multicentered cohort study with a control group. At each center, patients were first recruited into the control group and then into the intervention group. This nonrandomized design was chosen to avoid contamination bias caused by inevitable interaction between patients of the two groups and learning effects among the health care professionals. The study was approved by the ethics committee of the Medical Association of North Rhine, Germany and registered at the German Clinical Trials Register (DRKS00000765).

### Pharmaceutical care intervention

Patients in the control group received their treatment according to standard practice in Germany. They did not have regularly scheduled appointments with a pharmacist and were not exposed to pharmaceutical care.

Patients in the intervention group had regular appointments with the pharmacist providing pharmaceutical care. The medication was systematically documented in order to check for potential drug–drug interactions. The intervention consisted of two major components: the application of an algorithm for evidence-based antiemetic prophylaxis and treatment and medication counseling of the patients before and during their courses of chemotherapy.

The algorithm was developed by multidisciplinary consensus. The pharmacist drafted a tentative algorithm based on current evidence-based guidelines which served as a basis for discussion with the participating oncologists. During this discussion, the algorithm was modified until a consensus was reached. After implementation, the physicians chose the antiemetic prophylaxis for the patients of the intervention group according to the algorithm. Each prescription was checked by the pharmacist for adherence to the algorithm. Modifications were proposed if required. However, each physician had the final decision responsibility on whether the prescription was modified or not.

In addition, the patients were counseled regarding the optimal use of the supportive medication. Patients received general oral and written information about chemotherapy, potential adverse effects, and preventative strategies. In particular, patients were informed by the pharmacist about the antiemetic treatment, the importance of consequent prophylactic drug intake, the use of rescue medication, the mode of administration, and the correct dosing.

### Endpoints

“Complete response (CR) emesis,” defined as no emetic episode on the 5 days following chemotherapy, was the primary endpoint of the study. It was measured using a patient diary developed by Freidank [13], which covered both the acute and delayed phases of nausea and vomiting. Patients were asked to report the emetic episodes and classify the experienced nausea from degree 0 to 4 (“no nausea” to “severe nausea, which makes everyday life impossible”). Nausea and vomiting were documented after each cycle of chemotherapy in order to allow longitudinal evaluation of the data.

The severity of nausea and the frequency of vomiting were measured as secondary endpoints using the sum scores of the reported degrees of nausea and the number of emetic events, respectively, divided by the number of documented cycles in the acute and delayed phases.

Another secondary endpoint was health-related quality of life. It was measured with the validated German version of the cancer-specific EORTC QLQ-C30 (version 3.0) questionnaire [14] before the beginning, in the middle (after the second cycle for patients receiving four cycles of chemotherapy or after the third cycle for patients receiving six cycles of chemotherapy), and at the end of treatment (after the fourth or sixth cycle, respectively). For the second and third measurement, patients were asked to complete the QLQ-C30 questionnaire 1 week after chemotherapy administration. The scale values of the questionnaire were calculated according to the scoring manual provided by the EORTC [15]. Then, the absolute changes in the different scales over the treatment period compared to baseline were calculated, after reassuring that there were no differences between the groups at baseline.

Patient satisfaction with information on cancer treatment was measured as a secondary endpoint using the Canadian PS-CaTE questionnaire which had been translated into German and tested for its psychometric properties before [16]. Patients were asked to fill in the questionnaire after the last chemotherapy cycle. The questionnaire can be divided into four subscales and one global scale. The scale values were calculated as described by Liekweg et al. [16].

### Sample size calculation

The sample size calculation was performed by power simulation for the primary endpoint because, so far, no algorithms or programs are available to calculate the sample size for nonparametric analysis of variance. For this purpose, with different combinations of the group size (*n*), the prevalence in the control group (*p*), and the expected difference (∆), 10,000 simulations were performed at a time and the power was calculated for a type 1 error *α* = 5%. The simulations were performed by S-Plus® 2000. With a sample size of *n* = 50 per group and a prevalence of CR emesis of 40% to 60% in the control group, an improvement of ∆ = 15% in the intervention group can be detected with a power (1 − *β*) of more than 99%.

### Statistical methods

SPSS version 12.0 and SAS version 9.1 were used for the statistical analyses. In order to evaluate the primary endpoint “CR emesis,” the two groups were compared using the Kaplan–Meier analysis and the log-rank test as well as Fisher’s exact test for individual cycles. For the other endpoints, Mann–Whitney *U* test was used to compare both groups. As this was a longitudinal analysis with multiple measurements over time, a nonparametric factorial analysis for repeated measurements was performed to compare endpoints between groups over time, using treatment group as whole plot factor and time as split plot factor [17]. This analysis is based on ranks and assumes that measurements within one group at the same time point follow the same unspecified distribution. Measurements within one person over time may be correlated; however, no correlation structure was specified. *p* values <0.05 were considered statistically significant.

Moreover, a logistic regression was performed using aprepitant treatment and pharmaceutical care as independent variables in order to distinguish the influence of both interventions on CR emesis. The results are displayed with odds ratios, their 95% confidence intervals, and the *p* value.

## Results

### Patient population

Forty-eight patients of the control group and 50 patients of the intervention group were included in the analysis. The demographic characteristics of the patient population at baseline are presented in Table 1.

**Table 1:** Demographic characteristics of patient population

Sociodemographic characteristics	Control group (*n* = 48)	Intervention group (*n* = 50)	*p* value
Mean (SD) age (years)	54.4 (11.4)	49.6 (11.2)	0.047
Sex			0.742
Female	47 (97.9)	50 (100.0)	
Male	1 (2.1)	0	
Diagnosis			0.482
Breast cancer	43 (89.6)	47 (94.0)	
Ovarian cancer	5 (10.4)	3 (6.0)	
Chemotherapy regimen			<0.001
EC	23 (47.9)	10 (20.0)	
FEC	6 (12.5)	15 (30.0)	
EC-T	0	9 (18.0)	
EC-Doc	2 (4.2)	8 (16.0)	
AC	3 (6.3)	0	
FEC-Doc	0	2 (4.0)	
PC	5 (10.4)	3 (6.0)	
Not known	9 (18.8)	3 (6.0)	
Marital status			0.401
Married/partner	36 (75.0)	43 (86.0)	
Single	3 (6.3)	3 (6.0)	
Divorced	4 (8.3)	4 (8.0)	
Widow	4 (8.3)	0	
No answer	1 (2.1)	0	
Self-aid group			0.347
Yes	3 (6.3)	1 (2.0)	
No	44 (91.7)	49 (98.0)	
No answer	1 (2.1)	0	

*EC* epirubicin, cyclophosphamide; *FEC* fluorouracil, epirubicin, cyclophosphamide; *EC-T* epirubicin, cyclophosphamide, paclitaxel; *EC-Doc* epirubicin, cyclophosphamide, docetaxel; *AC* doxorubicin, cyclophosphamide; *FEC-Doc* fluorouracil, epirubicin, cyclophosphamide, docetaxel; *PC* paclitaxel, carboplatin

### Complete response emesis

**Fig. 1:**
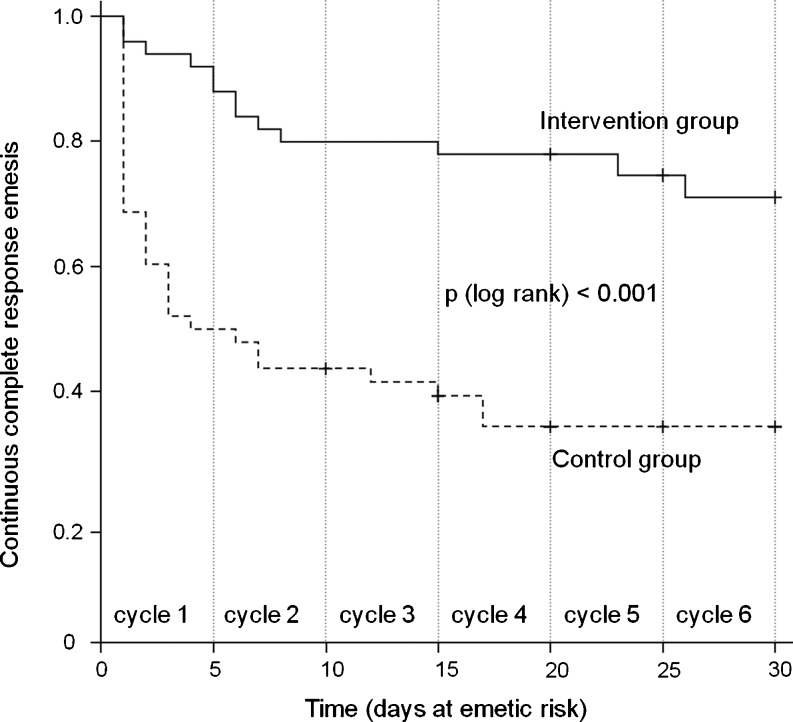
Kaplan–Meier estimates of continuous “CR emesis” (only days of emetic risk are shown, which were defined as the first 5 days of a cycle)

Pharmaceutical care led to a significant improvement of the primary endpoint “CR emesis” during the first four cycles of chemotherapy (Table 2). The nonsignificant results in cycles 5 and 6 are probably due to the small number of patients receiving these cycles. The longitudinal nonparametric analysis of variance according to Brunner confirmed the cyclewise results with a *p* value <0.001. The Kaplan–Meier curves in Fig. 1 also demonstrate the significantly better antiemetic outcome in the intervention group for the continuous “CR emesis” vs. days at emetic risk (log-rank, *p* < 0.001). Over the whole period, 35.4% (17 of 48) of patients in the control group had a CR compared to 76.0% (38 of 50) in the intervention group (*p* < 0.001). Thus, pharmaceutical care led to an absolute risk reduction for experiencing emetic episodes of 40.6%, which associates with a number needed to treat of 3.

**Table 2:** Number of patients with or without “CR emesis” (in percent)

	Control group			Intervention group			*p* value
	*n*	CR	No CR	*n*	CR	No CR	
Cycle 1	47	23 (48.9)	24 (51.1)	48	44 (91.7)	4 (8.3)	<0.001
Cycle 2	46	29 (63.0)	17 (37.0)	49	43 (87.8)	6 (12.2)	0.008
Cycle 3	44	27 (61.4)	17 (38.6)	48	45 (93.8)	3 (6.2)	<0.001
Cycle 4	40	24 (60.0)	16 (40.0)	48	46 (95.8)	2 (4.2)	<0.001
Cycle 5	14	11 (78.6)	3 (21.4)	27	26 (96.3)	1 (3.7)	0.107
Cycle 6	12	9 (75.0)	3 (25.0)	26	24 (92.3)	2 (7.7)	0.301

### Severity of nausea and frequency of vomiting

In the intervention group, the median severity of acute nausea was reduced by 39.4% compared to the control group. Median severity of delayed nausea was reduced by 34.5%. However, the differences between groups were not statistically significant. In contrast, there was a significant reduction in the frequency of emetic episodes (Table 3).

**Table 3:** Severity of nausea and frequency of vomiting

	Control group			Intervention group			*p* value
	*n*	Median	Quartiles	*n*	Median	Quartiles	
Nausea							
Acute	47	3.3	0.3; 10.3	47	2.0	0.0; 5.0	0.131
Delayed	48	11.9	0.8; 27.2	49	7.8	0.4; 19.7	0.324
Vomiting							
Acute	47	0.3	0.0; 1.5	49	0.0	0.0; 0.0	<0.001
Delayed	48	0.1	0.0; 0.5	49	0.0	0.0; 0.0	0.002

### Quality of life

The EORTC QLQ-C30 questionnaire is divided into global health as superior measure as well as functioning and symptom scales. Table 4 shows the absolute changes in quality of life from baseline to end of chemotherapy, respectively, for all scales. In particular, global health, social functioning, as well as the symptom scales “nausea and vomiting,” and “appetite loss” were positively affected by pharmaceutical care. When the absolute change was calculated only from baseline to the middle of chemotherapy the scale “pain” was also significantly affected (see Online resource 1). The longitudinal evaluation over time showed significant differences for the symptom scales “pain” (*p* = 0.023) and “appetite loss” (*p* = 0.009).

**Table 4:** Absolute change of quality of life from baseline to the end of chemotherapy

	Control group			Intervention group			*p* value
	*n*	Median	Quartiles	*n*	Median	Quartiles	
Global health	37	−8.3	−29.2; 0	49	0	−16.7; 12.5	0.020
Functioning scales							
Physical functioning	36	−6.7	−25.0; 5.0	46	0	−13.3; 8.3	0.145
Role functioning	34	0	−33.3; 16.7	49	0	−33.3; 16.7	0.496
Emotional functioning	34	−65.4	−81.7; −45.3	48	−64.3	−81.3; −47.3	0.799
Cognitive functioning	38	0	−16.7; 16.7	49	−16.7	−16.7; 0	0.169
Social functioning	39	0	−33.3; 16.7	49	0	−8.3; 16.7	0.058
Symptom scales							
Fatigue	37	11.1	0; 55.6	49	22.2	0; 33.3	0.540
Nausea and vomiting	38	33.3	12.5; 50.0	49	16.7	0; 33.3	0.003
Pain	36	0	−16.7; 16.7	47	0	−33.3; 16.7	0.276
Dyspnea	36	16.7	0; 33.3	49	0	0; 33.3	0.940
Insomnia	37	0	0; 33.3	49	0	−16.7; 33.3	0.368
Appetite loss	39	33.3	0; 66.7	49	0	−16.7; 33.3	0.010
Constipation	38	33.3	0; 66.7	49	0	0; 33.3	0.100
Diarrhea	38	0	0; 0	49	0	0; 0	0.763
Financial difficulties	38	0	0; 33.3	47	0	0; 0	0.254

### Patient satisfaction with information

The PS-CaTE questionnaire can be divided into four subscales (satisfaction with information on cancer treatment, side effects, complementary treatment options, and satisfaction with information sources) and global satisfaction as superior measure. Patient satisfaction with information was significantly improved upon pharmaceutical care (Table 5). The median values on a five-point Likert scale (1 = very dissatisfied to 5 = very satisfied) could be improved by 10% for cancer treatment, 7.5% for side effects, 5% for complementary treatment options, 12.5% for information sources, and 12.8% for global satisfaction (all calculated as relative changes), respectively. Except for the complementary treatment options scale, all improvements were statistically significant (Table 5).

**Table 5:** Patient satisfaction with cancer treatment education

	Control group			Intervention group			*p* value
	*n*	Median	Quartiles	*n*	Median	Quartiles	
Cancer treatment	41	4.0	3.7; 4.2	49	4.4	4.0; 4.8	0.017
Side effects	41	4.0	3.1; 4.3	49	4.3	4.0; 5.0	0.002
Complementary treatment options	41	4.0	3.0; 4.3	46	4.2	3.0; 5.0	0.145
Information sources	41	4.0	3.8; 4.8	49	4.5	4.0; 5.0	0.005
Global satisfaction	41	3.9	3.5; 4.2	49	4.4	3.8; 4.8	0.009

### Influence of aprepitant

During the course of the study, aprepitant was introduced for the prevention and treatment of nausea and vomiting and was integrated into the standard guidelines. Due to the sequential design, a higher proportion of patients of the intervention group were treated with aprepitant compared to the control group (35 of 50 vs. 5 of 47 patients). In order to discriminate between the influence of aprepitant and pharmaceutical care, a logistic regression analysis was performed using the inclusion method.

The model revealed a statistically significant influence of pharmaceutical care on CR emesis, whereas treatment with aprepitant showed no significant influence (*p* = 0.003 vs. *p* = 0.670). The odds ratio for the influence of pharmaceutical care was 5.4 (95% CI, 1.8–16.5) compared to 1.2 (95% CI, 0.5–3.3) for treatment with aprepitant. Thus, patients have a fivefold higher chance of showing a CR when changing from control to intervention group, and treatment with aprepitant did not bias the results to a significant extent.

## Discussion

Pharmaceutical care in oncology aims at reducing treatment-associated toxicity and at improving patients’ quality of life. During this project, a specific pharmaceutical care model for breast and ovarian cancer patients was implemented, including optimization of supportive medication and patient counseling on the management of treatment-associated adverse effects. The study revealed a benefit for the patients receiving pharmaceutical care based on improved PROs.

### Strengths and limitations

The major strength of this study is that it was entirely conducted under real-life conditions. A control group design was selected as the most suitable and generally accepted method. However, a number of limitations have to be considered before interpreting the data. Besides the relatively small number of patients, a nonrandomized study design was chosen. Since pharmaceutical care must be regarded as a highly complex intervention, some limitations with regard to the study design had to be accepted [18]. A parallel design with randomization could have led to significant contamination bias because of the interaction that occurs between clinic patients. In oncologic practices and outpatient clinics, patients have many possibilities of meeting each other and talking about the procedures and quality of care provided. It would have been impossible to avoid that patients of the control group would have noticed that other patients receive more information and attention than they do. Since especially cancer patients increasingly demand attention from health care professionals, a stringent refusal of pharmaceutical care to some of the patients would have been neither practical nor ethical. In addition, since blinding is not possible with this intervention, health care professionals might have adopted an approach to patient follow-up counseling that was dependent upon the intervention received. Therefore, we decided to recruit first the control group and subsequently the intervention group in each participating center.

Using a sequential enrolment instead of randomization led to some differences between the control and the intervention group that might potentially have influenced the results. First of all, the control group had a median age of 54.4 years compared to 49.6 years in the intervention group. Studies demonstrated that younger age is associated with a higher risk of vomiting [19]. Thus, the significantly better “CR emesis” in the intervention group was not positively biased by this difference. The second major difference can be found in the treatment regimens that have been used. Since the patients were approached consecutively, the observed difference in drugs and dosages is simply random and partly caused by the prior recruitment of the control group. Whereas the majority of the control group was treated with a combination of two drugs (54.2%), the majority of the intervention group received a combination (30%) or sequential treatment (34%) of three drugs. However, in both groups, the chemotherapy regimens were all classified as “moderately emetogenic” based on evidence-based guidelines [20]. Therefore, the emetic risk can be regarded as comparable in both groups.

### Nausea and vomiting

The primary endpoint “CR emesis” was significantly improved in the intervention group (35.4% of the control group vs. 76.0% of the intervention group, *p* < 0.001). This improvement can be accounted to the different components of the intervention. First of all, the intervention included the suggestion of a standardized, evidence-based antiemetic prophylaxis. Second, the intervention aimed at improving patients’ knowledge and discernment in the therapy and thus enhancing the concordance to the suggested prophylaxis. Especially for patients receiving a moderately emetogenic chemotherapy, antiemetic guidelines are often not applied [21] and implementation of guidelines into daily clinical practice is difficult [22].

The main difference of our algorithm to the previous practice in the participating centers was the reinforcement of a prophylactic antiemetic treatment and the evidence-based prevention of delayed emesis. Prior to the pharmacists’ intervention, the antiemetic treatment for the delayed phase was prescribed on demand. In addition, corticosteroids were rarely used and there was a widespread use of 5-HT_3_ antagonists for the prevention of delayed emesis. However, since this study was an observational study, it was not mandatory for treating physicians to follow the proposed algorithm for antiemetic prophylaxis and some deviations were still observed. Some physicians were reluctant to prescribe oral dexamethasone treatment on days 2 and 3 of the first cycle and only added dexamethasone in subsequent cycles of chemotherapy if the patient had major problems with nausea and vomiting. 5-HT_3_ antagonists were further used instead, even though this treatment has only limited efficacy in the prevention of delayed emesis. One could speculate that the consequent adherence to the guidelines would have resulted in even higher rates of CR in the intervention group and a more cost-effective treatment as dexamethasone is substantially less costly than 5-HT_3_ antagonists [23]. Nevertheless, even though physicians deviated from the agreed antiemetic algorithm in some patients of the intervention group, the majority received a guideline-conforming prophylaxis which can be regarded as improvement compared to the control group.

One major difference between the control group and the intervention group was the use of the NK1 receptor antagonist aprepitant. This drug was introduced during the course of the study and was rapidly implemented into international treatment guidelines for chemotherapy-induced nausea and vomiting [20]. Due to the later recruitment, a significantly larger proportion of patients of the intervention group was treated with aprepitant compared to the control group (35 vs. 5 patients). Therefore, we wanted to explore whether the intervention itself and not the use of aprepitant was responsible for the observed difference between the intervention group and the control group. The logistic regression performed showed that treatment with aprepitant as influencing factor alone did not result in statistically significant differences between the control group and the intervention group, whereas pharmaceutical care had a significant influence and resulted in an about fivefold increase of “CR emesis.” Comparing the results of the intervention group with the results of a study evaluating the efficacy of aprepitant in breast cancer patients receiving moderately emetogenic chemotherapy, the latter patient group showed a “CR emesis” of 51% [24] compared to 76% in our study. This supports the conclusions from the logistic regression that the improvement of the intervention group is also a result of pharmaceutical care and not only the use of aprepitant. However, the question remains whether pharmaceutical care is still effective when NK1 antagonists are widely used. In a recent study in two German university hospitals, a structured nursing intervention did not result in a significant reduction of nausea and emesis. The authors conclude that the impact of information and counseling programs on acute and delayed nausea and emesis might be limited when antiemetics are properly used [25]. Future studies will have to clarify this aspect.

In contrast to vomiting, patients of the intervention group did not show significant improvement regarding the severity of nausea both in the acute and delayed phases. A trend towards better outcomes in the intervention group could be observed; however, this did not reach statistical significance.

### Quality of life

Health-related quality of life includes physical, psychological, social, and functional dimensions [26]. The results for quality of life showed that global health as a determinant of overall quality of life was significantly improved in the intervention group. Furthermore, symptom scales such as “appetite loss” and “nausea and vomiting” showed significantly better results in the intervention group. These symptoms are closely linked to the antiemetic outcome. Improvement in these symptom scales are an indicator for a better overall quality of life [27]. Quality of life as a multidimensional construct is subject to large variability and various influencing factors. Different personalities (e.g., optimistic or pessimistic) as well as coping strategies affect quality of life substantially [28–31]. Therefore, with the limited patient number in our study, it was difficult to observe statistically significant differences.

### Patient satisfaction with information

Significant improvements in the intervention group were measured for the global scale and all subscales of the PS-CaTE questionnaire, except for information regarding complementary treatment options. These results demonstrate that the pharmaceutical care model may help increase the knowledge of the patients on different aspects of their treatment such as side effects. In contrast, patients were not actively informed on options for complementary treatments as evidence-based recommendations are lacking, which might explain the nonsignificant results for this scale. In general, information needs of cancer patients change with the course of their treatment [32]. For example, patients who were just recently diagnosed look for information on efficacy of treatment, potential side effects, supportive strategies, and consequences for their family life [33]. The advantage of our pharmaceutical care model is that it follows a needs-based approach with regard to patient information.

### Further considerations

When interpreting the data, one has to keep in mind that social effects may have influenced the data. It can be observed that patients who participate in studies regardless of the treatment and whether they are assigned to the control or intervention group seem to benefit. The manner in which patients are cared for in terms of emotional and cognitive care can influence the treatment outcome [34]. It might be interesting to investigate the relevance of such “context effects” on the outcome of pharmaceutical care in appropriate studies.

### Outlook

This study was conducted to explore the feasibility and the potential of a pharmaceutical care model by measuring PROs. With the study design selected, it was, however, not possible to fully evaluate the effectiveness of pharmaceutical care for cancer patients. Nevertheless, our study showed that pharmaceutical care models may help improve the quality of cancer care and are worth being investigated in larger trials involving a higher number of participating centers. In this case, patients in different oncologic outpatient clinics or practices could be randomly allocated to a particular intervention. Such “cluster randomized trials” are one solution to the problem of contamination of the control group and are increasingly used to evaluate complex interventions in health care [18, 35]. Moreover, the cost–benefit ratio of pharmaceutical care should be assessed in future studies in order to enforce the implementation of this intervention in clinical routine. Further studies and advanced activities in this area will certainly strengthen multidisciplinary and intersector collaboration in daily routine, which is urgently warranted to enhance patient safety in cancer therapy.

## Conclusions

In conclusion, our results suggest that pharmaceutical care for patients with breast and ovarian cancer is feasible and may have an impact on PROs as particularly indicated by significant improvements of the antiemetic response and patient satisfaction. Although there is no doubt that a higher awareness of drug-related problems is beneficial, final conclusions on the effectiveness of a new health care intervention can only be drawn when studied in a randomized trial. Therefore, our data may serve as a valuable basis for planning a large randomized multicenter trial.

## Electronic supplementary material

Below is the link to the electronic supplementary material.

Online resource 1 (PDF 12 kb)
