# Supplementary material for: Pharmaceutical care for patients with breast and ovarian cancer
Source: Support Care Cancer. 2012 Feb 2;20(11):2669–77. doi: 10.1007/s00520-012-1385-z (PMC3461211; doi:10.1007/s00520-012-1385-z)
Supplement: Supplementary file 1 — (PDF 12 kb) [file 520_2012_1385_MOESM1_ESM.pdf]

**Online Resources**

Title: Pharmaceutical Care for Patients with Gynaecological Malignancies

Journal: Supportive Care in Cancer

Authors: Andrea Liekweg, Martina Westfeld, Michael Braun, Oliver Zivanovic, Tania Schink, Walther Kuhn, Ulrich Jaehde

Affiliations: Department of Clinical Pharmacy, University of Bonn, An der Immenburg 4, 53121 Bonn, Germany; Center for Integrated Oncology (CIO), Department of Obstetrics and Gynaecology, University of Bonn, Sigmund-Freud-Str. 25, 53127 Bonn, Germany; Department of Clinical Epidemiology, Bremen Institute for Prevention Research and Social Medicine, Achterstr. 30, 28359 Bremen, Germany

Corresponding author**:**

Prof. Dr. Ulrich Jaehde, Institute of Pharmacy, Clinical Pharmacy**,** University of Bonn, An der Immenburg 4, D-53121 Bonn

Tel.: +49-228-73 5252, Fax: +49-228-73 9757, E-mail: u.jaehde@uni-bonn.de

Online resource 1: Absolute change of quality of life from baseline to the middle of chemotherapy

| | Control group | | | Intervention group | | | |
|---|---|---|---|---|---|---|---|
| | **n** | **Median** | **Quartiles** | **n** | **Median** | **Quartiles** | **p value** |
| **Global Health** | 44 | -12.5 | -28.1; 0 | 50 | 0 | -16.7; 8.3 | 0.007 |
| **Functioning scales** | | | | | | | |
| Physical functioning | 42 | 0 | -20.0; 1.7 | 48 | 0 | -13.3; 6.7 | 0.457 |
| Role functioning | 43 | 0 | -33.3; 0 | 48 | 0 | -29.2; 16.7 | 0.579 |
| Emotional functioning | 41 | 0 | -16.7; 20.8 | 48 | 8.3 | -8.3; 25.0 | 0.214 |
| Cognitive functioning | 43 | 0 | -16.7; 0 | 49 | 0 | -33.3; 0 | 0.473 |
| Social functioning | 44 | 0 | -16.7; 0 | 49 | 0 | -16.7; 16.7 | 0.004 |
| **Symptom scales** | | | | | | | |
| Fatigue | 41 | 22.2 | 11.1; 44.4 | 50 | 11.1 | 0; 44.4 | 0.264 |
| Nausea and Vomiting | 45 | 16.7 | 0; 50.0 | 50 | 16.7 | 0; 16.7 | 0.033 |
| Pain | 43 | 0 | 0; 33.3 | 50 | 0 | -33.3; 0 | 0.008 |
| Dyspnoea | 43 | 0 | 0; 33.3 | 50 | 0 | 0; 33.3 | 0.944 |
| Insomnia | 45 | 0 | 0; 33.3 | 49 | 0 | -33.3; 0 | 0.266 |
| Appetite loss | 46 | 33.3 | 0; 66.7 | 50 | 0 | 0; 33.3 | 0.005 |
| Constipation | 43 | 0 | 0; 66.7 | 49 | 0 | 0; 50.0 | 0.642 |
| Diarrhoea | 44 | 0 | 0; 0 | 50 | 0 | 0; 0 | 0.811 |
| Financial difficulties | 45 | 0 | 0; 33.3 | 48 | 0 | 0; 0 | 0.012 |
